# Toxicity of Metal Oxides, Dyes, and Dissolved Organic Matter in Water: Implications for the Environment and Human Health

**DOI:** 10.3390/toxics12020111

**Published:** 2024-01-28

**Authors:** Haradhan Kolya, Chun-Won Kang

**Affiliations:** Department of Housing Environmental Design, Research Institute of Human Ecology, College of Human Ecology, Jeonbuk National University, Jeonju 54896, Jeonbuk, Republic of Korea; hdk@jbnu.ac.kr

**Keywords:** toxicity, metal oxides, dissolved organic matter, dyes, water, aquatic lives, environments, human health

## Abstract

This study delves into the critical issue of water pollution caused by the presence of metal oxides, synthetic dyes, and dissolved organic matter, shedding light on their potential ramifications for both the environment and human health. Metal oxides, ubiquitous in industrial processes and consumer products, are known to leach into water bodies, posing a significant threat to aquatic ecosystems. Additionally, synthetic dyes, extensively used in various industries, can persist in water systems and exhibit complex chemical behavior. This review provides a comprehensive examination of the toxicity associated with metal oxides, synthetic dyes, and dissolved organic matter in water systems. We delve into the sources and environmental fate of these contaminants, highlighting their prevalence in natural water bodies and wastewater effluents. The study highlights the multifaceted impacts of them on human health and aquatic ecosystems, encompassing effects on microbial communities, aquatic flora and fauna, and the overall ecological balance. The novelty of this review lies in its unique presentation, focusing on the toxicity of metal oxides, dyes, and dissolved organic matter. This approach aims to facilitate the accessibility of results for readers, providing a streamlined and clear understanding of the reported findings.

## 1. Introduction

Water is a vital resource for sustaining life, and its quality is paramount for the well-being of ecosystems and human populations. However, in recent decades, increasing industrialization, urbanization, and agricultural practices have led to the release of various pollutants into water bodies, posing significant threats to aquatic ecosystems and human health [1]. Among these pollutants, metal oxides, dyes, and dissolved organic matter have emerged as notable concerns due to their diverse sources and potential toxicity [2].

Metal oxides, arising from both natural geological processes and human activities, impact water bodies through pathways like runoff, industrial discharges, and atmospheric deposition [3]. While well-known hazardous elements such as lead, mercury, cadmium, chromium, and arsenic pose risks, the spectrum extends to diverse metal oxides from 3d, 4d, and 5d transition metal series [4]. In the 3d series, iron oxides (Fe_2_O_3_, Fe_3_O_4_), copper oxides (CuO, Cu_2_O), and manganese oxides (MnO_2_) contribute to water pollution via natural weathering and anthropogenic activities like industrial emissions and urban runoff [5]. Additional contributors like nickel oxide (NiO), zinc oxide (ZnO), selenium oxides, and titanium oxide (TiO_2_) further emphasize the complexity of metal oxide pollution [6,7]. Expanding to the 4d and 5d transition metals, ruthenium oxides (RuO_2_) [8], rhodium oxides (Rh_2_O_3_), tantalum oxides (Ta_2_O_5_), and tungsten oxides (WO_3_) introduce diverse chemical characteristics from industrial processes, raising concerns about their ecological impacts [9]. Accumulation of these metal oxides in aquatic ecosystems disrupts environmental balance, impacting fish, plankton, and benthic organisms [10]. Bioaccumulation raises concerns about transference through the food chain, posing risks to human health [11]. This interplay underscores the need to understand metal oxides’ sources, behavior, and toxicological impacts on aquatic ecosystems [12]. A multifaceted approach is crucial for developing effective strategies to monitor, mitigate, and prevent the deleterious effects of these contaminants on water quality and human health.

Dyes, commonly used in industries like textiles, leather, and paper, contribute to water pollution by discharging effluents containing unutilized dyes [13]. The vibrant and diverse colors associated with dyes may be visually appealing, but their environmental impact is a cause for concern [14]. Many dyes utilized across diverse industries exhibit a concerning characteristic—persistence and resistance to degradation [15]. These pose substantial challenges for wastewater treatment plants and significantly heighten the risk of bioaccumulation in aquatic organisms [16]. Among the myriad of persistent dyes, several notable examples underline the severity of this issue [17]. Reactive dyes, known for their chemical ability to bond with fibers, exemplify persistence in the textile industry [18].

Similarly, direct dyes employed in the dyeing of cellulose fibers include notorious persisters like congo Red [19], direct blue 1, direct black 38, and direct blue 95 [20]. Disperse dyes, favored for synthetic fibers like polyester, introduce compounds such as disperse yellow 3 [21], disperse yellow 7 [22], disperse red 1 [23], and disperse orange 1 [24], demonstrating resistance to degradation. The azo dyes category, marked by azo bonds, boasts examples like orange II, sudan I, and acid red 88—known for their stability and persistence [25]. Vat dyes, commonly used in cotton dyeing [26,27], feature resilient options like indigo, benzanthrone, and anthraquinone [28,29]. Triarylmethane dyes, including malachite green and crystal violet, exhibit persistence, particularly in aquaculture applications [30]. The persistence of these dyes in wastewater streams necessitates advanced treatment methods, as traditional approaches often struggle to degrade these compounds effectively [31]. This persistence challenges the efficiency of wastewater treatment and raises the bioaccumulation specter in aquatic organisms. The consequences of this resistance to degradation extend beyond environmental concerns, encompassing potential ecological imbalances and posing risks to human health [32].

Dissolved organic matter (DOM), a diverse blend of organic compounds which passes through a 0.45 μm filter, is pivotal in shaping water quality within aquatic ecosystems [33,34]. While a natural component, human-driven activities such as deforestation, agriculture, and urbanization amplify its presence in water bodies [35]. The spectrum of DOM includes various components such as humic acids, derived from the decomposition of organic matter, microplastics, fulvic acids, tannins, amino acids, and plant-derived sugars [36,37]. These compounds not only contribute to the color of water but also engage in intricate interactions with metal oxides [38]. The formation of complexes between DOM and metal oxides can lead to alterations in toxicity, impacting the overall health of aquatic ecosystems [39]. Humic and fulvic acids, for instance, are known for forming complexes with metals, influencing their bioavailability [40]. Recognizing the diversity of DOM components is crucial for understanding the nuanced dynamics of these interactions, emphasizing the need for sustainable practices to manage and mitigate the repercussions on water quality and ecological balance [41].

While previous studies have often examined these pollutants in isolation, understanding their synergistic effects is essential for comprehensively assessing water quality. Metal oxides, arising from both natural processes and human activities, can interact with synthetic dyes and DOM, forming complex chemical reactions with potential ecological consequences [39]. In addition, the coexistence of metal oxides, synthetic dyes, and dissolved organic matter in irrigation water engenders a complex web of interactions with profound implications for soil properties. Metal oxides, originating from natural processes and anthropogenic activities, introduce variations in soil pH and influence the solubility and precipitation of other pollutants [42]. Synthetic dyes, known for their persistence, can alter the soil’s chemical composition, affecting nutrient availability and microbial activity [43]. Concurrently, dissolved organic matter, a complex mixture of organic compounds, interacts with metal oxides, potentially forming complexes that impact soil structure and nutrient cycling [44,45]. The interplay of these pollutants in irrigation water may trigger intricate reactions, influencing the overall soil environment. Understanding the implications of metal oxides, dyes, and dissolved organic matter in water is crucial for comprehending the complex interplay between contaminants and their effects on the environment and human health [46,47]. This review aims to explore the current knowledge regarding these pollutants’ sources, distribution, and toxicological impacts, shedding light on the potential risks they pose to aquatic ecosystems and human health. By showcasing the toxicity aspects of these contaminants, the review enhances the usability of the information, making it more readily applicable for those seeking insights into the environmental and health impacts of metal oxides, synthetic dyes, and dissolved organic matter. The elucidation of the ecological disruptions caused by metal oxides, coupled with the persistent challenges of synthetic dyes and the variable toxicity of dissolved organic matter, underscores the intricate dynamics influencing both environmental integrity and human health. The unique focus on showcasing the toxicity aspects enhances the practical usability of the reported findings, making them more accessible for researchers.

## 2. Source of Metal Oxides and Toxicity

**Figure 1:**
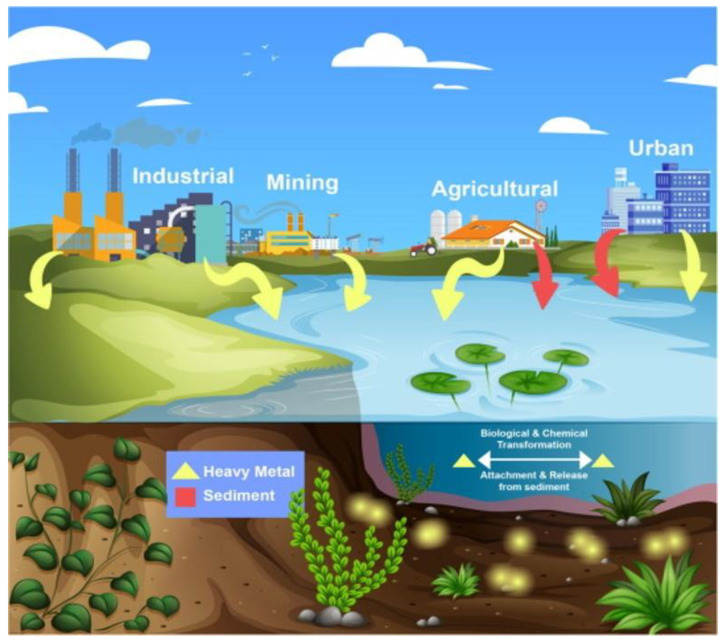
Diagrammatic illustration of toxic metals in the environment [48]. Copyright 2022, reproduced with permission from Elsevier B.V.

**Figure 2:**
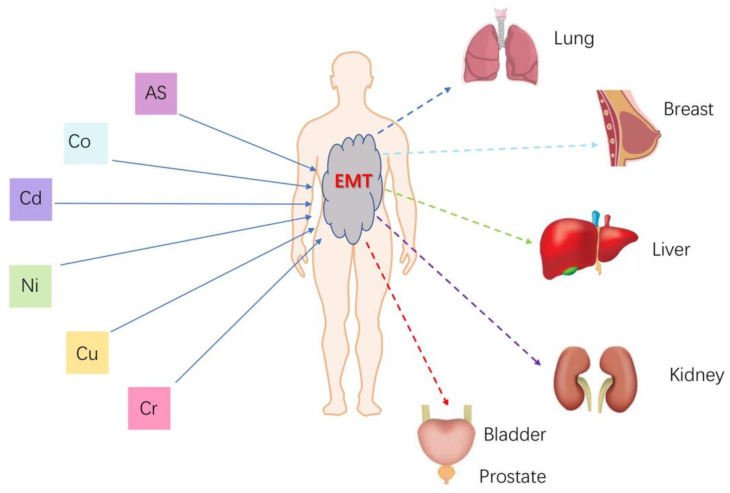
Metal ion toxicity in humans [72]. Copyright 2022, reproduced with permission from Springer Nature.

Metal oxide contaminants infiltrate the environment through various pathways, showcasing the intricate interplay of natural and anthropogenic processes. Erosion of rocks and minerals contributes to the release of metal ions, while industrial activities discharge metal oxides into waterways [36]. Agricultural runoff carries metal-based fertilizers into water bodies, impacting aquatic ecosystems. Urban runoff from corroded structures adds to water pollution. Mining operations disturb soil, releasing metal oxides into rivers. Natural disasters mobilize metal oxides, affecting water quality. Atmospheric deposition introduces metals into ecosystems, and improper waste disposal in landfills perpetuates metal persistence. Biological processes contribute to metal accumulation, and intentional use of metal-based materials in applications like adsorbents and nanoparticles introduces these contaminants. Recognizing these diverse sources is crucial for implementing effective measures to safeguard water quality and environmental well-being. A summary of the sources (Figure 1) and effects of toxic metal oxides on human health (Figure 2) and aquatic life is presented in Table 1.

Additionally, the toxicity findings for several metal oxides vary, and the available information on some compounds is limited. Palladium oxide (PdO) is relatively inert, and while specific toxicity data are limited [65], respiratory and skin irritation precautions are advised. Iridium oxides (IrO_2_) have limited toxicity data [66], but as with many metal oxides, careful handling is necessary to prevent respiratory hazards. Osmium tetroxide (OsO_4_) is highly toxic, causing severe respiratory and skin irritation, eye damage, and its use is confined to controlled laboratory settings [67]. Platinum oxides (PtO_2_) have limited specific toxicity information, but occupational exposure should be managed to prevent potential cytotoxic issues [68]. Hafnium oxides (HfO_2_) have limited information on cytotoxicity [69], and precautions are recommended during handling. Rhenium oxides (ReO_3_) and niobium pentoxide (Nb_2_O_5_) have limited toxicity data, with rhenium and niobium considered to have low toxicity [70,71]. Comprehensive studies are needed to fully understand exposure to these metal oxides’ potential health and environmental implications. Occupational safety measures, including proper handling and exposure prevention, remain crucial in industrial settings.

The metal oxides listed (Table 1) exhibit common adverse effects on human health and aquatic life. Neurological impacts, respiratory issues, and carcinogenicity are recurring concerns for human health, emphasizing the potential health risks associated with exposure. In aquatic ecosystems, these metal oxides threaten fish, invertebrates, and plants, disrupting the balance of ecosystems. Bioaccumulation, respiratory irritation, and ecological toxicity are shared consequences, highlighting the need for comprehensive management strategies to mitigate these contaminants’ impact on human and environmental well-being.

## 3. Source of Dyes and Toxicity

**Figure 3:**
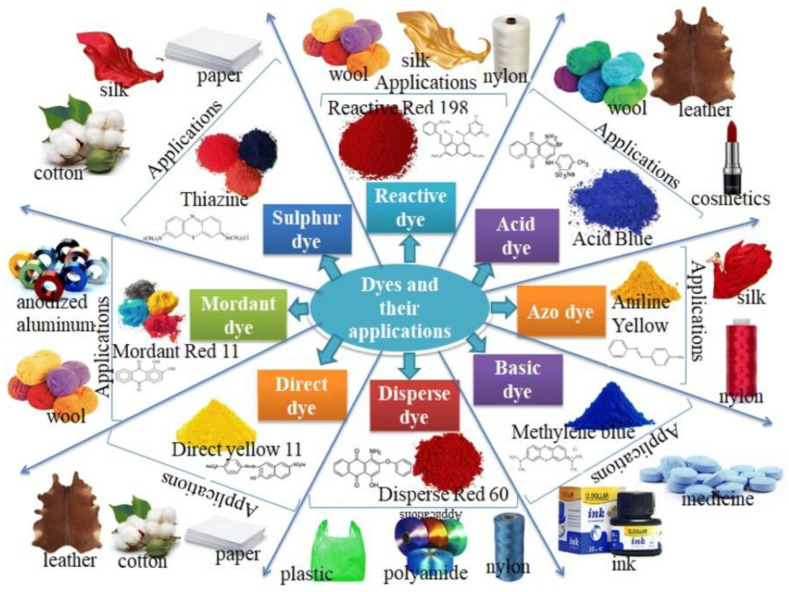
Exploring various dye varieties and their diverse industrial applications [79]. Copyright 2023, reproduced with permission from the Royal Society of Chemistry (RSC); RSC Publishing; Cold Spring Harbor Laboratory Press.

Synthetic dyes encompass diverse types, each designed for specific applications [73]. Acid dyes, known for vibrant hues, find purpose in coloring wool and silk and staining bacteria and yeast [74,75]. Basic dyes with cationic properties excel in dyeing synthetic fibers like acrylic [76]. Disperse dyes are the go-to for coloring polyester and acetate textiles. Reactive dyes form durable bonds with fibers, making them a staple in textile dyeing. Vat dyes, prized for colorfastness, are ideal for dyeing cotton. Direct dyes are versatile, easy to apply, and suitable for natural fibers like cotton and wool. Sulfur dyes, cost-effective and deep in color, are favored for dyeing cotton. Metal complex dyes offer intense colors and resistance to fading in applications such as textiles and inks. Azo dyes [77], a diverse category, find use in textiles, plastics, and printing inks for their vivid hues [78]. Solvent dyes bring vibrant coloration to non-polar solvents like plastics and waxes. This diversity underscores these dyes’ specialized roles in various industries, contributing to the vibrant spectrum of colored products in our daily lives (Figure 3).

**Figure 4:**
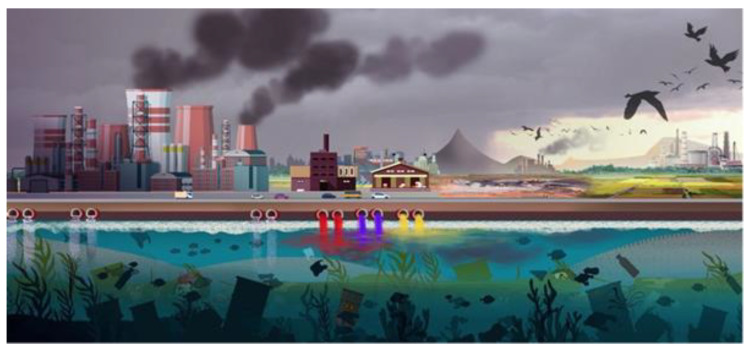
Schematic representation of water pollution originating from industrial effluents [80]. Copyright 2021, reproduced with permission from John Wiley and Sons.

Hence, the primary source of dyes in water arises from untreated industrial effluents discharged into water bodies. Additionally, a non-negligible source of dyes will be domestic washing of clothes. This is a common task at home or in neighborhood laundries and is an activity in which the washing water goes directly into the sewage system. It is worth mentioning this situation and even trying to assess whether the loss of dye, for example, from some denim jeans during successive washing, loses more dye than in their production process. Textile dyeing processes pose a significant environmental threat, with up to 15% of applied dyes escaping wastewater. The extensive use of water in dyeing procedures results in continuous improper discharge. Even after treatment, dye effluents contain high levels of pollutants, including toxic metals, chlorinated compounds, and organic substances. This persistent contamination adversely affects air, soil, plants, and water resources, contributing to severe human health issues [73]. A summary of the sources (Figure 4) and the effects of toxic dyes on human health and aquatic life is presented in Table 2.

Table 2 summarizes various synthetic dye types and their effects. Ionic dyes cause skin and respiratory issues, impacting fish and invertebrates. Nonionic disperse dyes have low acute toxicity but potential carcinogenic risks, affecting aquatic plants. Nonionic vat dyes exhibit low toxicity with limited impact on aquatic life. Cationic dyes induce skin irritation and respiratory issues, potentially harming fish and aquatic plants. Anionic acid and direct dyes lead to skin irritation and respiratory problems, adversely affecting fish, invertebrates, and aquatic invertebrates. Anionic reactive dyes induce skin irritation and respiratory issues, impacting aquatic plants. This underscores synthetic dyes’ diverse toxic impacts on human health and aquatic ecosystems. Moreover, textile dyes, even at low concentrations, pose environmental hazards due to their non-biodegradable nature. Contaminated water can lead to health issues like skin rashes, headaches, nausea, and, notably, an increased cancer risk. The toxicity of these dyes is categorized into acute and chronic/genotoxic effects, with the latter posing long-term health risks. Reactive dyes, commonly used, are associated with skin irritation and various allergic reactions [80].

## 4. Source of Trace Toxic Organic Pollution

**Figure 5:**
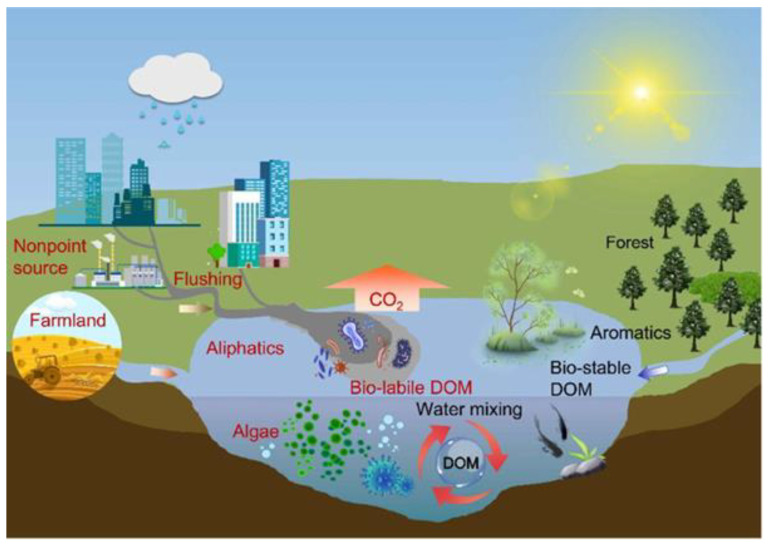
Schematic illustration of the source of dissolved organic matter [88]. Copyright 2022, reproduced with permission from the American Chemical Society.

Dissolved organic matter (DOM) or trace toxic organic matters in water originates from diverse natural and human-induced sources [88]. Natural sources include decomposing organic materials such as dead plants, animals, and microorganisms [89]. Soil runoff, facilitated by rainfall or irrigation, carries organic compounds from the land into water bodies [90]. Leaching from plant materials, like leaves and branches, adds to the dissolved organic content influenced by natural ecosystems and human activities like deforestation [91]. Wastewater discharges from domestic or industrial sources introduce organic matter into water, and the decomposition of organic pollutants further contributes to trace toxic organic pollution [92]. Urban runoff, stemming from rainwater washing over impervious surfaces, also contributes to the organic load in water bodies. Aquatic plants and algae release organic compounds through natural processes, and atmospheric deposits, including airborne particles and plant material, can settle into water through precipitation (Figure 5) [88]. Understanding the multifaceted origins of dissolved organic matter is essential for comprehending water quality dynamics and ecosystem health. A summary of the sources (Figure 5) and the effects of trace toxic organic matters on human health and aquatic life is presented in Table 3.

The toxicity of DOM in human health and aquatic life varies depending on the specific compound. While DOM is a natural component of aquatic ecosystems, human-induced activities contribute significantly to its increased presence [121]. The interactions between DOM and metal oxides, as well as other contaminants, can lead to the formation of toxic complexes, impacting the overall health of aquatic ecosystems [122]. Understanding the sources and toxicological effects of DOM is crucial for effective water quality management and the protection of both human and environmental health. The listed organic compounds (Table 3), sourced from industrial processes and runoff, exhibit diverse toxic effects on human health and aquatic life. Common concerns include carcinogenicity, respiratory issues, neurological effects, and endocrine disruption. The compounds impact fish, invertebrates, and aquatic ecosystems, with varied health consequences across species. The potential for bioaccumulation and disruption of aquatic ecosystems underscores the need for a comprehensive understanding and management of these contaminants.

## 5. Discussion

This comprehensive review has significantly advanced our comprehension of the intricate dynamics associated with metal oxides, synthetic dyes, and trace toxic organic matter in aquatic ecosystems [123,124]. By delving into the multifaceted challenges posed by these contaminants, the review underscores the urgent need for a holistic approach to water quality management. The elucidation of the ecological disruptions caused by metal oxides, coupled with the persistent challenges of synthetic dyes and the variable toxicity of dissolved organic matter (DOM) or trace toxic organic matter, highlights the complex interplay influencing both environmental integrity and human health. The review’s unique emphasis on showcasing the toxicity aspects enhances the practical usability of the reported findings, rendering them more accessible for researchers, policymakers, and practitioners alike. Moreover, the call for targeted research and the development of innovative water treatment technologies addresses crucial research gaps, paving the way for more effective and sustainable solutions [46]. The integration of these vital components lays the groundwork for making informed decisions in the fields of both environmental and public health.

## 6. Conclusions

This review illuminates the multifaceted challenges associated with metal oxides, synthetic dyes, and dissolved organic matter or trace toxic organic matter in water ecosystems. The intricate interplay of contaminants, ranging from transition metals to persistent synthetic dyes and complex organic compounds, underscores the critical need for a holistic understanding of their sources and toxicological impacts. The ecological disruption caused by metal oxides, the challenges posed by the persistence of synthetic dyes, and the varied toxicity of trace toxic organic matter necessitate nuanced approaches to water quality management. The review emphasizes the importance of adopting multidisciplinary strategies, encompassing effective monitoring, mitigation measures, and preventive actions, to safeguard environmental integrity and human health. Additionally, the authors propose targeted research to address gaps in understanding contaminant interactions and the development of innovative technologies for water treatment. This paper could emphasize the need for concerted efforts in research, policy development, and practical interventions to ensure the sustainable preservation of water resources.

## Figures and Tables

**Table 1:** Sources and effects of toxic metal oxides on human health and aquatic life.

Metal Oxides	Source	Toxicity in Human Health	Toxicity in Aquatic Life	Refs.
Lead oxide (PbO)	Industrial processes, mining	Neurological effects, developmental issues	Adverse effects on fish and invertebrates	[47]
Mercury oxide (HgO)	Coal combustion, mining	Neurological damage, kidney damage	Bioaccumulation in fish, toxic to invertebrates	[49]
Cadmium oxide (CdO)	Smelting, battery production	Kidney damage, bone effects	Toxic to aquatic organisms, disrupts ecosystems	[50]
Arsenic oxide (As_2_O_3_)	Mining, agricultural runoff	Skin lesions, cancer risk	Toxic to fish, invertebrates, and plants	[51,52]
Nickel oxide (NiO)	Metal refining, combustion	Respiratory issues, carcinogenic, infertility	Toxic to aquatic invertebrates	[53]
Zinc oxide (ZnO)	Mining, industrial processes	Gastrointestinal issues	Adverse effects on fish and aquatic plants	[54]
Selenium oxides	Mining, agricultural runoff	Selenosis, liver damage	Toxic to fish and aquatic invertebrates	[55]
Titanium oxide (TiO_2_)	Paints, sunscreens, industrial use	Limited health risks (in nanoparticle form)	Low toxicity, but environmental concerns	[56]
Copper oxide (CuO)	Mining, agricultural pesticides	Gastrointestinal issues, liver damage	Toxic to fish, invertebrates, and aquatic organism	[57]
Chromium oxide (Cr_2_O_3_)	Metal plating, leather tanning	Respiratory issues, carcinogenic	Toxic to aquatic organisms, bio accumulative	[58]
Vanadium pentoxide (V_2_O_5_)	Metal smelting, fuel additives	Respiratory and cardiovascular issues, cytotoxic	Toxic to fish, invertebrates, and aquatic plants	[59]
Manganese oxides (MnO_2_)	Mining, industrial processes	Neurological effects, respiratory issues	Adverse effects on fish and aquatic invertebrates	[60]
Ruthenium oxides (RuO_2_)	Anthropogenic activities, metal refining, electronics	Respiratory irritation and, in extreme cases, lung damage	Potential ecotoxicological effects	[60]
Rhodium oxides (Rh_2_O_3_)	Precious metal refining, automotive catalysis, and electronics manufacturing	Irritate the respiratory tract, and, like many metal oxides	Ecotoxicological impacts in high concentration	[61]
Tantalum oxides (Ta_2_O_5_)	Electronics, capacitors, metallurgical operations	Teratogenic, respiratory irritation, and reproductive toxicity	Limited information is available on the specific effects of tantalum oxides on aquatic life	[62,63]
Tungsten oxides (WO3)	Metal manufacturing, alloys	Cytotoxic, respiratory irritation, and genotoxic	Eco-toxicological effects of tungsten oxides are not extensively studied	[64]

**Table 2:** Sources and effects of toxic dyes on human health and aquatic life.

Synthetic Dye Type	Source	Toxicity in Human Health	Effects on Aquatic Life	Refs.
Ionic dyes	Industrial processes, textile dyeing	Skin irritation, respiratory issues	Toxic to fish, invertebrates	[81]
Nonionic (disperse): Disperse red 1 Disperse blue 1	Textile dyeing, plastics, polyesters	Low acute toxicity, but potential carcinogenic	Adverse effects on aquatic plants	[82]
Nonionic (vat): Indanthrene blue rs Vat yellow 2	Textile dyeing, printing inks, plastics	Low toxicity, limited health risks	Limited impact on aquatic life	[83]
Cationic dyes: Crystal violet, methylene blue, basic blue 9, and malachite green	Textile dyeing, paper industry	Skin and eye irritation, respiratory issues	Potential toxicity to fish and aquatic plants	[83,84]
Anionic (acid): Acid orange 7 Acid red 73 Acid blue 9 Acid red 52 Acid black 1	Textile dyeing, leather tanning, paper industry	Skin irritation, respiratory issues	Adverse effects on fish, invertebrates	[85]
Anionic (direct): Direct blue 6 Direct blue 86 Direct red 81 Direct yellow 4 Direct black 19	Textile dyeing, paper industry, leather tanning	Skin irritation, respiratory issues	Toxic to aquatic invertebrates	[20,86]
Anionic (reactive): Reactive blue 19 Reactive red 120 Reactive yellow 145 Reactive black 5	Textile dyeing, printing, paper industry	Skin irritation, respiratory issues	Adverse effects on aquatic plants	[87]

**Table 3:** Sources and effects of trace toxic organic matters on human health and aquatic life.

Organic Compound	Source	Toxicity in Human Health	Toxicity in Aquatic Life	Refs.
Polycyclic Aromatic Hydrocarbons	Combustion of fossil fuels, industrial processes	Carcinogenic, respiratory, and reproductive issues	Toxic to aquatic bodies	[93,94]
Pesticides (e.g., atrazine)	Agricultural runoff, urban runoff	Neurological effects, endocrine disruption	Impacting insects and fish	[95]
Benzene	Industrial discharges, urban runoff	Carcinogenic, respiratory, and central nervous system issues	Toxic to aquatic organisms, affecting fish and insects	[96]
Chlorinated Compounds	Industrial discharges, atmospheric deposition	Neurological, renal, and developmental issues	Impacting fish and invertebrates	[97]
Volatile Organic Compounds	Industrial emissions, vehicle exhaust	Teratogenic, carcinogenic, mutagenic, genetic neurotoxicant and toxicant	Affecting fish and microorganism	[98]
Dioxins and Furans	Combustion of waste, forest fires, volcanic eruptions	Respiratory irritation, skin disorders, liver problems, cancer	Fish skin diseases	[99,100]
Phthalates	Industrial discharges, plastic leaching, migration, and oxidation	Neurotoxic and genotoxic	Toxic to water bodies animals	[101]
Nonylphenol	Industrial discharges, shampoos, detergents	Endocrine disruption, developmental issues	Impacting fish	[102]
Endocrine Disruptors	Industrial discharges, wastewater treatment, climate change, cosmetics	Endocrine disruption, reproductive issues, and hormonal imbalance	Affects fish embryonic development	[103]
Pharmaceuticals and Personal Care Products	Household wastewater, improper disposal	Variable health effects	Variable effects on aquatic organisms	[104,105]
Per- and Polyfluoroalkyl Substances	Industrial processes, firefighting foam, metal coating, wastewater treatment plants	Neurobehavioral toxicity, developmental effects, metabolism abnormalities	Bioaccumulation and toxic effects on aquatic organisms	[106]
Cyanobacterial Toxins	Harmful algal blooms, nutrient runoff	Liver damage, potential, carcinogenic effects	Affecting freshwater bodies	[107]
Nitrosamines	Agricultural runoff, wastewater treatment, combustion process, domestic source	Carcinogenic potential, potential reproductive issues	Toxic to aquatic and terrestrial organisms	[108,109]
Trihalomethanes	Chlorination of drinking water	Carcinogenic potential, reproductive, thyroid hormone endocrine functions issues	Algae toxicity, fragile aquatic ecosystem	[110]
Acrylamide	Industrial processes, wastewater treatment plants	Neurotoxic effects, carcinogenic, reprotoxic, and mutagenic	Toxic marine fish	[110]
Organophosphate Flame Retardants	Textile manufacturing, electronics production, wood protection	Neurological effects, potential endocrine disruption	Affecting fish reproduction	[111]
Polybrominated Diphenyl Ethers	Flame retardants in electronics, textiles	Neurodevelopmental issues, potential carcinogenicity, and immune toxicity	Alters thyroid hormone levels and gene transcription of fish	[112]
Polychlorinated Biphenyls	Industrial processes, improper disposal	carcinogenicity, hormone disruption, neurodevelopmental toxicity	Bioaccumulation in fish, dolphins, crabs	[113]
Bisphenol A	Plastics, epoxy resins, can linings	Endocrine disruption, potential reproductive issues	Health damage in fish	[114,115]
Chlorophenols	Organic synthesis, industrial wastewater, wood industries	Respiratory, dermatological effects, mutagenicity, endocrine-disrupting potency	Affecting fish and bioaccumulation potential in fish tissues	[116]
Diethylhexyl Phthalate	Plasticizers in plastics, consumer products	Endocrine disruption, potential reproductive issues	Altered the antioxidant system in the liver, intestine, brain, and gills	[117,118]
Glyphosate (Herbicide)	Agricultural runoff, urban runoff	Potential carcinogenicity, endocrine disruption, cytotoxicity	Impact on fish framing	[119,120]
